# Introduction

**DOI:** 10.1186/1471-2105-14-S1-I1

**Published:** 2013-01-14

**Authors:** Riccardo Rizzo, Paulo JG Lisboa

**Affiliations:** 1 ICAR-CNR, National Research Council of Italy, Viale delle Scienze Ed. 11, Palermo, 90128, Italy; 2 School of Computing and Mathematical Sciences, Byrom Street, Liverpool John Moores University, Liverpool L3 3AF, UK

## 

The International Meeting on Computational Intelligence Methods for Bioinformatics and Biostatistics (CIBB) is a small but active conference where a group of researchers share their ideas and visions. The proceedings of the conference are usually published in a book and a group of selected papers are collected in a post proceedings book published by Springer.

In 2010 the 7^th ^CIBB conference was hosted in Palermo at Palazzo Comitini during September 16-18. We found several of the papers to merit further development and publication as full journal articles to achieve the broader diffusion that they deserve. The result is this *BMC Bioinformatics *supplement. All of the papers published in this supplement are completely new and a further advancement of the research presented in the CIBB meetings. Moreover the goal of this supplement is not only to present the research of the participants of the CIBB meeting but to promote the CIBB conference to the world bioinformatics community.

The CIBB meeting series is organized by the Special Interest Group on Bioinformatics of the International Neural Networks Society (INNS) to provide a forum open to researchers from different disciplines to present and discuss problems concerning computational techniques in bioinformatics, systems biology and medical informatics with a particular focus on neural networks, machine learning, fuzzy logic, and evolutionary computational methods. From 2004 to 2007, CIBB meetings were held with an increasing number of participants in the format of a special session of bigger conferences, namely WIRN 2004 in Perugia, WILF 2005 in Crema, FLINS 2006 in Genoa and WILF 2007 in Camogli. With the great success of the special session at WILF 2007 that included 26 strongly rated papers, we launched the first autonomous CIBB conference edit starting with the 2008 conference in Vietri.

Riccardo Rizzo and Paulo JG Lisboa

## Competing interests

The authors declare that they have no competing interests.

## Declarations

This article has been published as part of *BMC Bioinformatics *Volume 14 Supplement 1, 2013: Computational Intelligence in Bioinformatics and Biostatistics: new trends from the CIBB conference series. The full contents of the supplement are available online at http://www.biomedcentral.com/bmcbioinformatics/supplements/14/S1.

